# Macro- and micromechanical remodelling in the fish atrium is associated with regulation of collagen 1 alpha 3 chain expression

**DOI:** 10.1007/s00424-018-2140-1

**Published:** 2018-03-28

**Authors:** Adam N. Keen, Andrew J. Fenna, James C. McConnell, Michael J. Sherratt, Peter Gardner, Holly A. Shiels

**Affiliations:** 1 Division of Cardiovascular Sciences, Manchester Academic Health Sciences Centre, University of Manchester, Manchester, UK; 2 Centre for Tissue Injury and Repair, Faculty of Biology, Medicine, and Health, University of Manchester, Manchester, UK; 3 School of Chemical Engineering and Analytical Science, Manchester Institute of Biotechnology, University of Manchester, Manchester, UK

**Keywords:** Compliance, Heart, Stiffness, Temperature acclimation, Phenotypic plasticity

## Abstract

Numerous pathologies lead to remodelling of the mammalian ventricle, often associated with fibrosis. Recent work in fish has shown that fibrotic remodelling of the ventricle is ‘reversible’, changing seasonally as temperature-induced changes in blood viscosity alter haemodynamic load on the heart. The atrial response to varying haemodynamic load is less understood in mammals and completely unexplored in non-mammalian vertebrates. To investigate atrial remodelling, rainbow trout were chronically cooled (from 10 ± 1 to 5 ± 1 °C) and chronically warmed (from 10 ± 1 to 18 ± 1 °C) for a minimum of 8 weeks. We assessed the functional effects on compliance using ex vivo heart preparations and atomic force microscopy nano-indentation and found chronic cold increased passive stiffness of the whole atrium and micromechanical stiffness of tissue sections. We then performed histological, biochemical and molecular assays to probe the mechanisms underlying functional remodelling of the atrial tissue. We found cooling resulted in collagen deposition which was associated with an upregulation of collagen-promoting genes, including the fish-specific collagen I alpha 3 chain, and a reduction in gelatinase activity of collagen-degrading matrix metalloproteinases (MMPs). Finally, we found that cooling reduced mRNA expression of cardiac growth factors and hypertrophic markers. Following long-term warming, there was an opposing response to that seen with cooling; however, these changes were more moderate. Our findings suggest that chronic cooling causes atrial dilation and increased myocardial stiffness in trout atria analogous to pathological states defined by changes in preload or afterload of the mammalian atria. The reversal of this phenotype following chronic warming is particularly interesting as it suggests that typically pathological features of mammalian atrial remodelling may oscillate seasonally in the fish, revealing a more dynamic and plastic atrial remodelling response.

**Electronic supplementary material:**

The online version of this article (10.1007/s00424-018-2140-1) contains supplementary material, which is available to authorized users.

## Introduction

Chronic changes in pressure or volume load can cause the vertebrate heart to change in size, form and function [57, 46]. This cardiac remodelling response is often compensatory and maintains optimal cardiac function under conditions of increased haemodynamic preload or afterload. Studies in mammals show chronic pressure or volume overload is associated with remodelling of both the atria and ventricle; however, atrial remodelling is less well understood [67, 60, 46]. Chronic atrial dilation (or atrial enlargement) is a form of cardiomegaly, which may be associated with cellular hypertrophy, myocardial fibrosis, angiogenesis, apoptosis and myolysis [3]. The increase in non-conducting extracellular matrix (ECM) can delay and/or impair electrical conduction between cardiomyocytes and may initiate alternate conduction pathways [71, 60]. This can contribute to atrial arrhythmias, atrial fibrillation (AF) and loss of contractility [55]. Symptoms can be self-perpetuating with atrial fibrillation contributing to atrial dilation and the occurrence and maintenance of fibrosis [67, 72, 41]. Enlargement of the atria may also result from increased load on the heart that occurs with increased physiological demand, such as exercise training [26, 59, 10]. However, this kind of ‘physiological’ remodelling gives a compensatory increase in myocardial wall thickness, which regresses when the stimulus is removed; it is not associated with arrhythmias or fibrosis and thus contrasts with ‘pathological’ remodelling [59].

The role of the atrial contraction in filling the mammalian ventricle is relatively small, providing ~ 20–30% of end-diastolic volume (depending on age) [24]. However, in fish, the single atrium directly modulates stroke volume of the single ventricle by acting as a volume reservoir for ventricular end-diastolic volume [18]. The fish heart consisted of four chambers in series, the sinus venosus which collects venous blood from the body, the atrium, the ventricle and the bulbus arteriosus which is the outflow track leading from the heart to the gill [28]. The fish atrium is the largest chamber of the heart and is formed from an external ring of myocardium with thin but highly trabeculated walls and a complex pectinate-like web of trabeculae which aid contractions by pulling the walls and roof inwards [15, 18]. Although debated [47, 1], it has been suggested that atrial systole is the primary mechanism for ventricular filling in fish, and therefore crucial for determining filling volume, strength of contraction and total stroke volume of the fish heart [32, 15].

Rainbow trout remain active throughout the year despite the direct effects of temperature on contractile force and blood viscosity, which can alter cardiac load [11, 35]. Indeed, increased blood viscosity during cooling has been suggested as the hypertrophic trigger for remodelling in fish hearts due to the increased haemodynamic stress of pumping viscous blood [8, 23]. Many studies have investigated temperature-induced remodelling of the fish ventricle [23, 39, 40, 13, 19, 36]. Sex, level of sexual maturation and circannual rhythms are known to influence the degree of ventricular hypertrophic remodelling in fish [8, 19, 39, 40]. However, studies investigating the role of the atrium in thermal remodelling in fish are very limited. Proteins involved in excitation-contraction coupling like SERCA, phospholamban, Ca^2+^-binding proteins and the Na^+^-Ca^2+^ exchanger show similar changes in gene expression following cold acclimation in the atrium compared with the ventricle [42, 43, 30]. Furthermore, certain proteins, such as FK506-binding protein which regulates sarcoplasmic reticulum Ca^2+^ release, are upregulated in the atrium and remain unchanged in the ventricle following chronic cooling [44]. Finally, there is evidence of partial positive thermal compensation, as atrial contraction kinetics improve following cold acclimation in trout [1]. Whether atrial fibrosis occurs following chronic cooling is currently unknown; however, significant ventricular fibrosis has been shown with cold acclimation in the rainbow trout [39, 35]. We know of no studies to directly investigate atrial remodelling following both warming and cooling in any fish species.

Here, we investigate the effects of chronic cooling (from 10 ± 1 to 5 ± 1 °C) and chronic warming (from 10 ± 1 to 18 ± 1 °C) on the rainbow trout atrium. These temperatures reflect those experienced seasonally by trout. We focus our study on changes in the passive properties of the atrium, across multiple levels of organisation. Based on our recent work on the trout ventricle [35], we hypothesised that chronic cooling would increase atrial stiffness and fibrosis and upregulate growth factors associated with pathological remodelling in mammals. We further hypothesise that the opposite would occur following chronic warming. To understand the functional consequences of thermal acclimation, we used atomic force microscopy (AFM) to test micromechanical atrial stiffness, generated ex vivo atrial pressure-volume curves to test whole chamber compliance and used in situ zymography to assess gelatinase activity of matrix metalloproteinases (MMPs). To determine structural remodelling of the tissue, we used histological stains to assess ECM proteins. We then used quantitative real-time PCR (RT-qPCR) to examine growth factors, collagen isoform expression, connective tissue regulators and hypertrophic markers following prolonged temperature exposures. As trout experience intermittent temperature change, we were particularly interested in variable remodelling between chronic warming and chronic cooling. Our findings suggest that chronic cooling increases cardiac preload, which causes chronic dilation of the atrium leading to a change in diastolic function. We found the opposite remodelling response following chronic warming, suggesting that atria remodel seasonally in fish.

## Materials and methods

### Ethical approval

All husbandry and housing conditions were in accordance with the local handling protocols and adhere to the UK Home Office legislation. All experimental procedures were approved by the University of Manchester’s ethical review committee.

### Experimental animals

Sexually mature female rainbow trout (*Oncorhynchus mykiss*; *n* = 47; morphometric data in Table 1) were purchased from Dunsop Bridge Trout Farm (Clitheroe, UK), housed on a 12-h light:12-h dark cycle in ~ 500-l re-circulated aerated fresh water tanks at 10 ± 1 °C and fed to satiation three times per week. Water quality was ensured with 30% water changes three times per week and regular tests for temperature, pH, ammonia, nitrates and nitrites. Fish were held under these conditions for a minimum of 2 weeks before being randomly assigned to one of three acclimation groups: cold (5 ± 1 °C), control (i.e. no change, 10 ± 1 °C) or warm (18 ± 1 °C). These temperatures were based on previous literature which describes the cardiac remodelling response in salmonids [35, 39]. Water temperature of the warm and cold acclimation groups was changed by 1 °C per day until desired temperature was reached and then held at that temperature for a minimum of 8 weeks before experiments. The photoperiod for the cold-acclimated animals was changed to 8-h light:16-h dark cycle to simulate winter [23].

**Table 1:** The gross morphological parameters of thermally acclimated rainbow trout

	Cold acclimated (5 °C)	Control (10 °C)	Warm acclimated (18 °C)
Mass (g)	480.8 ± 64.3	526.1 ± 42.8	524.8 ± 60.2
Heart mass (g)	0.94 ± 0.09	1.15 ± 0.12	0.99 ± 0.11
RHM (g mass^−1^ × 100)	0.21 ± 0.010	0.22 ± 0.0086	0.19 ± 0.0079
Atrial mass (g)	0.12 ± 0.013	0.13 ± 0.02	0.14 ± 0.017
RAM (g mass^−1^ × 100)	0.027 ± 0.0019	0.023 ± 0.0019	0.026 ± 0.0014

Values given are mean ± S. E. Significance was determined by GLM with Tukey post hoc test for comparison between the groups (*P* < 0.05), *n* = 18 for each group

*RHM* relative heart mass, *RAM* relative atrial mass

Before experiments, fish were stunned by a blow to the head followed by severance of the spinal cord and destruction of the brain by pithing. The heart was excised, rinsed in phosphate-buffered saline and weighed. Atria were used immediately for the ex vivo pressure-volume curves. Atria to be used for RT-qPCR were snap frozen and stored at − 80 °C. Atria to be used for histological analysis and in situ zymography were bisected down the sagittal plane with one half snap frozen in OCT (Thermo Fisher Scientific, Waltham, MA, USA) and stored at − 80 °C. The other half was fixed in 10% neutral buffered formalin solution (Sigma-Aldrich, St. Louis, MO, USA) before being processed and embedded in paraffin wax.

### Ex vivo passive pressure-volume curves

Whole chamber compliance was tested by generating ex vivo pressure-volume curves. The intact isolated heart was placed into an organ bath containing Ringer’s solution [(in mM) 150 NaCl, 5.4 KCl, 2.0 CaCl_2_, 1.5 MgSO_4_, 0.4 NaH_2_PO_4_, 10 HEPES, 10 glucose at a pH of 7.7 with NaOH at room temperature] at 10 ± 1 °C to which 20 mM BDM (2, 3 butanedione monoxime) was added to prevent active cross-bridge cycling. Pressure-volume curves from atria from each acclimation group were generated at a common temperature, 10 ± 1 °C, to isolate the effects of chronic remodelling on myocardial stiffness from the acute effects of temperature. A cannula was fed through the sinus venosus into the atrial lumen and secured at the sino-atrial junction, using 0-0 silk thread (Harvard Apparatus, Holliston, MA, USA). An atraumatic clamp was placed at the atrio-ventricular junction making the atrium a sealed chamber with the cannula inside. The cannula was connected to a syringe pump (INFORS AG, Bottmingen, CHE), in series with a pressure transducer, containing 10 ± 1 °C Ringer’s solution with BDM and a small amount of blue food colouring (Silverspoon, London, UK). Before filling commenced, while the atrium was empty, pressure in the atrium was manually set to 0 kPa. The pressure transducer was calibrated daily against a static water column and measurements recorded at 1000 Hz (Chart5, PowerLab, ADI Instruments, Dunedin, New Zealand). Ringer’s solution with BDM was pumped into the atrium at 0.05 ml min^−1^ until maximum volume was achieved, determined by visual leak of the saline-containing blue dye and a drop in the pressure trace.

### Atomic force microscopy

Atrial tissue micromechanics were tested using atomic force microscopy (AFM). Frozen atrial tissue was sectioned at 5 μm (Leica CM3050S cryostat, Leica, Wetzlar, Germany) and mounted onto microscope slides. Excess OCT was removed with distilled water and the slides were left to dry for ~ 12 h. This methodology is consistent with previous work [37, 82], which shows that tissue sections are best preserved, dehydrated with rehydration performed when nano-mechanical measurements are required. Micro-indentation was performed using a Bioscope Catalyst AFM (Bruker, Coventry, UK) mounted onto an Eclipse T1 inverted optical microscope (Nikon, Kingston, UK) fitted with a spherically tipped cantilever (nominal radius and spring constant of 1 μm and 3 Nm^−1^ respectively; Windsor Scientific Ltd., Slough, UK) running Nanoscope Software v8.15 (Bruker, Coventry, UK). The local reduced modulus (*E*_r_) was determined for each of 400 points in a 50 × 50 μm region, indented at a frequency of 1 Hz with lateral spacing of 2.5 μm. *E*_r_ is a measure of stiffness, which is similar to Young’s or elastic modulus, but which does not rely on an estimate of the Poisson ratio of the sample being tested. The extend curve was used in conjunction with a contact point-based model to calculate the *E*_r_ for each indentation [9]. For each biological sample, 400 force curves were collected at three distinct 50 μm^2^ regions. Once all 400 force curves had been generated, a quality control was applied where any force values falling more than two standard deviations away from the mean value were discarded in order to account for failed indents. Data loss at this stage was less than 10% (data not shown).

### Tissue histology

Tissue morphology was assessed histologically. Formalin-fixed and paraffin-embedded atrial tissue was sectioned at 5 μm using a microtome (Leica RM2255, Leica, Wetzlar, Germany), mounted onto glass slides (Superfrost Plus, Thermo Fisher Scientific, Waltham, MA, USA) and stained using haematoxylin and eosin (H&E). The preparations were not pressurised before fixation as the atrial tissue was bisected for use in histology and zymography. Previous studies have used myocyte bundle cross-sectional area as a proxy for myocyte cross-sectional area as single fish myocytes are too narrow (diameter of 3–6 μm) to resolve in cross section histologically [39, 35]. Cross-sectional bundle area and extra-bundular sinus (EBS) space were quantified using ImageJ software [70]. For morphometric analysis of myocyte bundle cross-sectional area and EBS space, eight sections were analysed per individual fish. For measurement of cross-sectional area of myocyte bundles, three separate image montages were taken along transects across the full diameter of the cross section on each tissue section. In each image, trabeculations were chosen for measurement only if they were in the transverse plane, i.e. the image showed a cross section of the trabeculations making it circular in appearance. For EBS space, the non-tissue area of each image was measured.

Fibrillar collagen and elastin content were analysed semi-quantitatively following previously published methodology [22, 35]. Briefly, formalin-fixed paraffin-embedded atrial tissue was sectioned at 5 μm (Leica RM2255 microtome, Leica, Wetzlar, Germany) and mounted onto glass slides. Serial sections from each sample were stained with picro-sirus red for collagen [33] and Miller’s elastic stain for elastin [52]. Picro-sirus red images were quantified using polarised light microscopy and Miller’s elastic images were quantified using bright-field microscopy. Mean fibrillar collagen content was expressed as a percentage of total tissue cross-sectional area, excluding the epicardial surface, determined using ImageJ. Three tissue sections were considered for each individual to ensure consistency in measurements. On each tissue section, three separate image montages were taken along transects across the full diameter of the cross section. All histological analysis was conducted blind to the acclimation group, and in all cases, these tissue sections were taken from a central portion of the atrial wall, away from either the sino-atrial or atrio-ventricular junctions.

### In situ MMP gelatin zymography

The activity of endogenous MMP gelatinase was semi-quantitatively analysed by in situ zymography of tissue cryosections, following previously published methodology [53, 2]. Frozen tissue was sectioned at 10 μm (Leica CM3050S cryostat, Leica, Wetzlar, Germany) and mounted onto glass slides (Superfrost Plus, Thermo Fisher Scientific, Waltham, MA, USA). Low-temperature gelling agarose (Sigma-Aldrich, St. Louis, MO, USA) was dissolved in phosphate-buffered saline (to a final concentration of 10 mg ml^−1^) in an 80 °C water bath and then cooled to 37 °C. DQ gelatin (porcine; Invitrogen, Thermo Fisher Scientific, Waltham, MA, USA) was dissolved in dH_2_O (to a concentration of 1 mg ml^−1^) and diluted 1:10 in the agarose solution. Lastly, 1 μg ml^−1^ 4′, 6′-diamidino-2-phenylindole (DAPI) was added. During this time, tissue sections were brought to room temperature and washed in PBS to remove excess OCT. Approximately 40 μl of agarose/DAPI/DQ gelatin was added to each tissue section and a coverslip placed on the slide to ensure even film thickness across the sample section. All samples were incubated in the dark for 1 h at 4 °C and then 18 h at room temperature. Following incubation, the samples were imaged immediately using a fluorescent microscope with a green filter (Leica, Wetzlar, Germany). To account for tissue auto-fluorescence, negative control slides were used to determine the microscope settings for each section. Three tissue sections were measured for each individual. On each tissue section, three separate image montages were taken along transects across the full diameter of the cross section. Following background subtraction, mean fluorescence intensity was calculated for each image and analysed using ImageJ. Analysis was conducted blind to the acclimation group, and in all cases, these tissue sections were taken from a central portion of the atrial wall, away from either the sino-atrial or atrio-ventricular junctions.

### Quantitative real-time PCR

Transcript abundance of genes associated with muscle growth (ventricular myosin heavy chain, VMHC; muscle LIM protein, MLP; and small myosin light chain 2, SMLC2), hyperplasia (proliferating cell nuclear antigen, PCNA), angiogenesis (vascular endothelial growth factor, VEGF), collagen I (Col1a1, Col1a2 and Col1a3), connective tissue regulators (MMP2, MMP9, MMP13 and TIMP2), stretch and heart failure markers (ANP and BNP) and a pro-hypertrophic nuclear factor of activating T (NFAT) signalling mediator (a regulator of calcineurin; RCAN1) were quantified in the atria of fish from cold-acclimated, control and warm-acclimated groups (*n* = 7 atria for each temperature). RNA was extracted from 5 mg of snap-frozen tissue (RNeasy Micro Kit, Qiagen, Venlo, NL) and amount and quality were determined (NanoDrop ND-1000, NanoDrop, Wilmington, DE, USA). An RNA concentration of 200 ± 50 ng μl^−1^ was used to make cDNA with SuperScript III First Strand Synthesis System (Invitrogen, Carlsbad, CA, USA). SYBR Green I pre-mixed chemo-technology was used for qPCR. qPCR was carried out in a 7900 HT sequence detection system (Applied Biosystems, Carlsbad, CA, USA). All primers were the same as those used in Keen et al. [35], which were designed using Primer 3 from mRNA sequences available on PUBMED. All expression levels were normalised to housekeeping gene *β*-actin to determine absolute expression levels for comparison at each acclimation temperature. Three housekeeping genes were tested, *β*-actin, GAPDH and DNAJ1, and *β*-actin had the most stable expression in relation to temperature acclimation, as found previously [31, 35]. Supplementary Table 1 contains the list of specific primers used for quantitative real-time PCR.

### Statistical analysis

Chamber filling volume was calculated from filling time by the equation:

$$ \mathrm{volume}\ \left(\mathrm{ml}\right)=\mathrm{time}\ \left(\upmu \mathrm{s}\right)\times \frac{0.05}{60}\times 1000 $$

The effect of temperature acclimation on the pressure-volume relationship was assessed by a general linear model (GLM) with pressure as the dependent variable, volume and acclimation group as fixed factors and body mass as the covariate, with a Tukey post hoc test for differences between groups, using R [62]. The output from GLM is provided in Supplementary Table 2. Differences in myocyte bundle cross-sectional area, EBS space, collagen deposition, gelatinase activity and transcript abundance were assessed by GLM with Holm-Sidak post hoc test for differences between groups using Prism v6 (GraphPad Software, Inc., La Jolla, CA, USA). Following conventional one-way ANOVA on the full distribution of the AFM data, post hoc analyses of AFM force curves were performed using Nanoscope Analysis v1.40 (Bruker, Coventry, UK), whereby a baseline correction was applied to each curve before a force fit was applied using a Herzian (spherical) model and a maximum force fit of 70%. For all analyses, significance was considered to be *P* < 0.05, except for atomic force curves where significance was considered at *P* < 0.005. Values are presented as mean ± S. E. throughout except for atomic force curves where values are mean ± S. D. Specifics of statistical tests are provided in the figure legends.

## Results

### Ex vivo chamber compliance

**Fig. 1:**
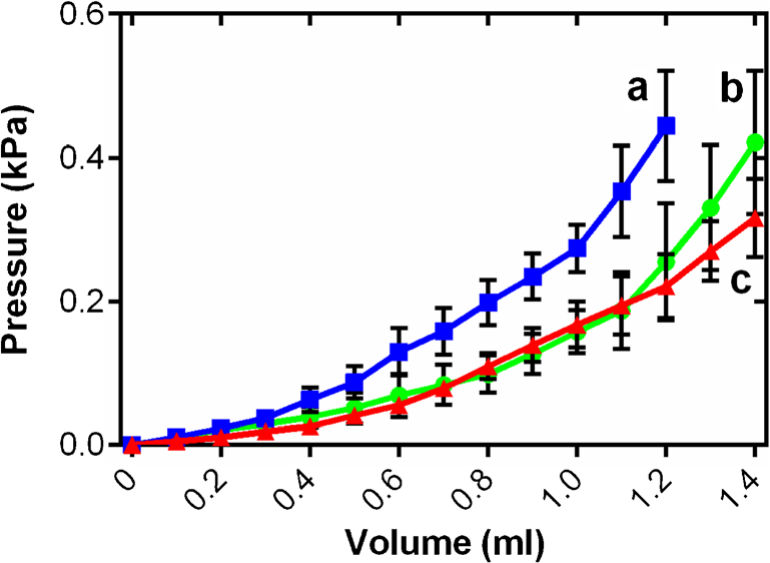
Ex vivo atrial passive filling pressure-volume relationships for cold-acclimated (5 °C; blue squares), control (10 °C; green circles) and warm-acclimated (18 °C; red triangles) rainbow trout (*n* = 8). Values are mean ± S. E., at all points on the curve *n* > 3. Pressure has been standardised to start at 0 kPa for graphical representation. These volumes and pressures encompass the physiological range. Significant differences in compliance between acclimation groups were assessed by a GLM with volume as the dependent variable, treatment and pressure as the fixed factors and chamber mass as the covariate (*P* < 0.05), shown by dissimilar letters. See Supplementary Table 2 for the statistical output which provides clarity on the differences demarked here

Chronic changes in cardiac load can influence chamber compliance [5, 35]. To study this in fish atria, we generated ex vivo passive filling curves from freshly isolated intact atria, treated with BDM, at a common test temperature of 10 °C to assess the functional effects of cardiac remodelling on the passive properties of the tissue. Atrial pressure increased exponentially with filling volume for each temperature acclimation group. Figure 1 shows the mean data for each acclimation temperature from 0 to the maximum physiological filling pressures experienced by rainbow trout in vivo; central venous pressure is usually below 0.1 kPa and maximal mechanical efficiency of the trout heart has been observed at a preload of ~ 0.3 kPa [16, 23, 18, 25]. Thermal acclimation altered the pressure-volume relationship during atrial filling (*R*^2^ = 0.63, *F*_2_, _20,335_ = 1328.3, *P* < 0.001) showing increased stiffness after chronic cooling, across all filling volumes, compared to control and warm tissue. Conversely, chronic warming caused a moderate increase compliance compared to controls at high filling volumes (*t* ratio = 40.5; Fig. 1). The details and interactions of the statistical model are given in Supplementary Table 2.

### Micromechanical tissue stiffness

**Fig. 2:**
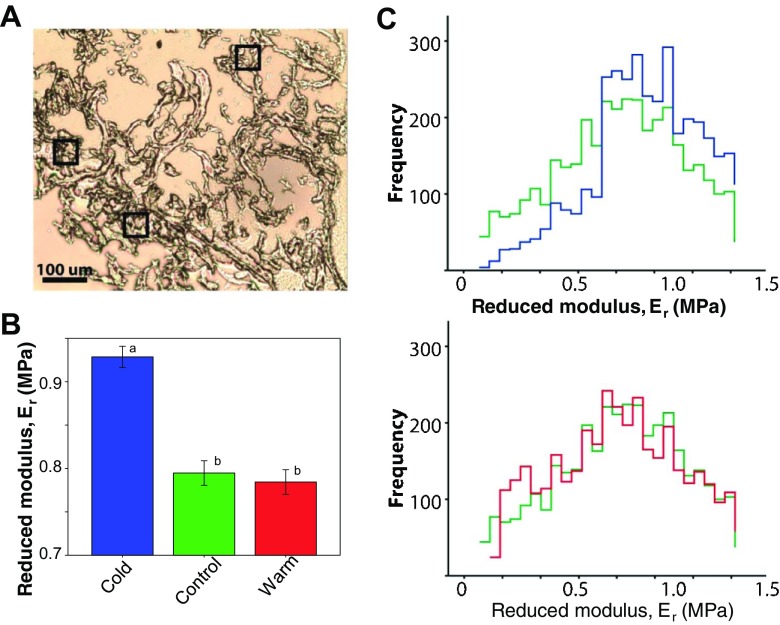
Atrial tissue micromechanics. **a** Unstained bright-field microscope image of a control atrial cryosection with black boxes (50 × 50 μm) demarking measurement areas. **b** Mean reduced modulus (*E*_r_) was influenced by acclimation temperature. Values presented are mean ± S. D. Significance was assessed by one-way ANOVA and is shown between groups by dissimilar letters (*P* < 0.005). **c** Frequency distribution of *E*_r_, in cold (5 °C; blue) versus control (10 °C; green) (top), and warm (18 °C; red) versus control (bottom). *n* = 3 animals per group, 3 regions per animal, 400 force curves per region. The frequency histograms are separately overlaid to show the spread of the data and to emphasise the differences between control and cold and between control and warm

As chamber stiffness is associated with the micromechanical properties in the heart [17], we used AFM nano-indentation of atrial cryosections to determine if chronic temperature exposure induced changes in local tissue stiffness. Figure 2a shows an unstained bright-field micrograph of a representative control section of fish atrium, with black boxes showing the size and location of experimental test areas. The sparse structure of the tissue is normal for fish atrium and its morphology is discussed more fully below. Mean reduced modulus (*E*_r_) was significantly higher in cold atrial tissue when compared with control and warm-acclimated tissue (*P* < 0.005; Fig. 2b), indicating that cold acclimation increased micromechanical stiffness. The frequency of *E*_r_ showed a standard distribution for each temperature (Fig. 2c), suggesting that tissue micromechanics remain homogenous with thermal acclimation. Clear differences in the frequency of *E*_r_ can be observed between control and cold atria across the range of *E*_r_ observed (Fig. 2c, top). More subtle are the differences in the frequency distribution between control and warm atria, which are limited to the lower range of *E*_r_ (Fig. 2c, bottom).

### The atrial extracellular matrix

**Fig. 3:**
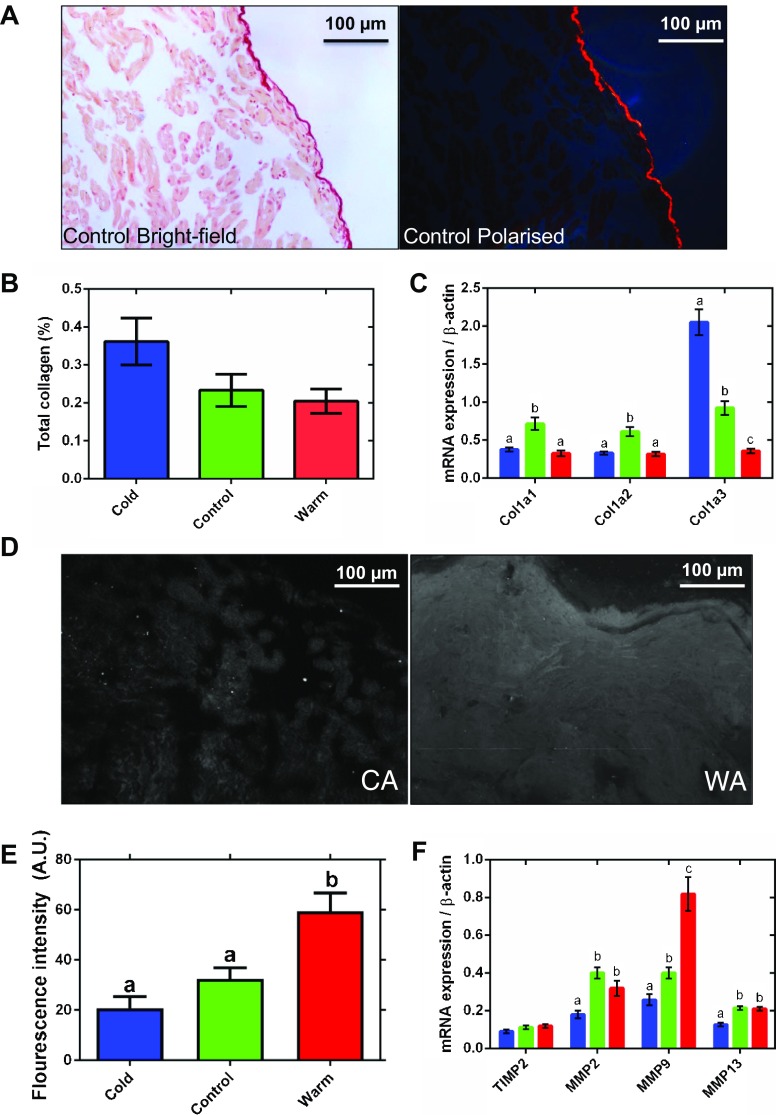
Atrial connective tissue remodelling. A representative control atrial tissue micrograph, imaged under **a** bright-field (left) and polarised (right) light, stained with picro-sirus red. **b** Semi-quantitative analysis of collagen content from polarised light images expressed as a percentage of total tissue. The epicardial surface of the tissue has been excluded from the analysis. There is a trend toward an increase in collagen in the cold (*P* = 0.063). **c** mRNA expression of collagen genes (Col1a1, Col1a2, Col1a3). **d** Representative fluorescent micrographs of tissue treated with DQ gelatin to show gelatinase activity of matrix metalloproteinases (MMPs) in white for cold-acclimated (CA, left) and warm-acclimated (WA, right) atrium. **e** Semi-quantitative analysis of gelatinase enzyme activity. **f** mRNA expression of collagen regulatory genes (TIMP2 = upregulation; MMP2, MMP9 and MMP13 = downregulation) for cold-acclimated (5 °C; blue), control (10 °C; green) and warm-acclimated (18 °C; red) rainbow trout (*n* = 10 fish per acclimation group; 3 replicates for each animal were averaged for both histology and qPCR). Values presented are mean ± S. E. Significance was assessed by GLM with a Tukey, or Holm-Sidak for multiple comparisons, post hoc test. Significance between groups is shown by dissimilar letters (*P* < 0.05). Supplementary Table 1 contains the list of specific primers used for quantitative real-time PCR

Changes in cardiac stiffness/compliance are associated with remodelling of the ECM in both fish and mammals [7, 39, 35]. We used picro-sirus red to assess fibrillar collagen content (Fig. 3a). Collagen fibres are visualised under plane polarised light and subtracted from the total tissue area. The highly collagenous epicardial surface, which is a feature of fish atrial tissue [28], was excluded from the analysis as its signal swamped the lower signals detected in the trabeculae. Percent collagen in trabeculae was low, and although there was a trend toward increased collagen content in cold-acclimated animals (*P* = 0.063), this was not statistically resolvable (Fig. 3b). We were able to detect elastin in vessels using Miller’s elastic histological staining but did not detect any penetrating into the atrial muscle under any thermal condition (not shown).

In mammals, collagen I accounts for ~ 80% of total collagen in the myocardium and is the main collagen in cardiac fibrosis [50]. Mammalian collagen I is composed of type 1 (α1) (col1a1) and type 2 (α2) (col1a2) alpha-helical chains; fish also have an additional type 3 (α3) chain (col1a3) [66]. mRNA expression of Col1a3 was 5.7-fold higher in the cold- compared to the warm-acclimated atrium (*P* < 0.05; Fig. 3c). Expression of the Col1a1 and Col1a2 mRNA was downregulated in both cold and warm fish compared with controls, suggesting temperature-independent remodelling is also occurring.

Matrix metalloproteinases (MMPs) regulate the ECM and, therefore, tissue collagen content [54]. We assessed gelatinase activity of MMPs in tissue sections by in situ zymography. Figure 3d shows a representative fluorescent micrograph for gelatinase activity in cold-acclimated (left) and warm-acclimated (right) atria. Semi-quantification of fluorescence intensity showed a 1.8-fold increase in gelatinase activity following warm acclimation compared to controls (*P* < 0.05); however, there was no difference between cold-acclimated and control groups (Fig. 3e). Most MMPs are gelatinases and our mRNA analysis showed that MMP2, MMP9 and MMP13 were 2.2-, 3.2- and 1.7-fold lower in the atrium of cold-acclimated animals than of warm-acclimated animals (*P* < 0.05; Fig. 3f), suggesting these MMPs may contribute to the decreased gelatinase activity following cooling which is evident in Fig. 3d. MMP activity is regulated by tissue inhibitors of MMPs (TIMPs), and thus, increased TIMP activity is associated with increased collagen deposition in tissue with active turnover. However, we found no change in expression of the trout atrial TIMP2 gene following prolonged temperature change (*P* < 0.05; Fig. 3f). We did not investigate other TIMPs in this study.

### Atrial muscle morphology

**Fig. 4:**
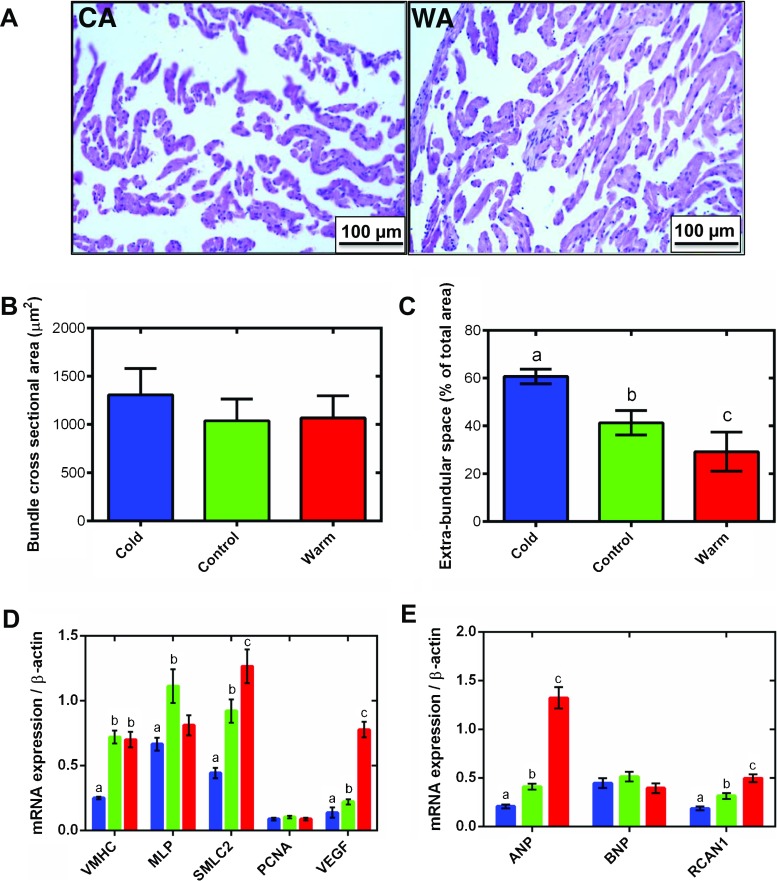
Atrial tissue remodelling. Representative haematoxylin and eosin (H&E)-stained atrial tissue sections for **a** cold-acclimated (CA, left) and warm-acclimated (WA, right) rainbow trout. Quantification of **b** myocyte bundle cross-sectional area and **c** extra-bundular sinus with temperature acclimation. For measurement of cross-sectional area of myocyte bundles, three separate image montages were taken along transects across the full diameter of the cross section on each tissue section. In each image, trabeculations were chosen for measurement only if they were in the transverse plane, i.e. the image showed a cross section of the trabeculations making it circular in appearance. For EBS space, the non-tissue area of each image was measured. **d** mRNA expression of markers of muscle growth (VMHC, MLP and SMLC2), hyperplasia (PNCA) and angiogenesis (VEGF). **e** Hypertrophic markers (ANP and BNP) and regulator of the pro-hypertrophic NFAT signalling pathway (RCAN1). In **b**–**e**, cold (5 °C; blue), control (10 °C; green) and warm (18 °C; red) acclimation. *n* = 10 fish per acclimation group; 3 replicates for each animal were averaged for both histology and qPCR. Values presented are mean ± S. E. Significance was assessed by GLM with Holm-Sidak post hoc test. Significance between groups is shown by dissimilar letters (*P* < 0.05). Supplementary Table 1 contains the list of specific primers used for quantitative real-time PCR

Atrial chamber size and wall thickness can alter myocardial compliance in accordance with the law of Laplace [34]; thus, we investigated aspects of gross atrial muscle morphology following thermal acclimation. Temperature acclimation did not affect total atrial mass or atrial mass relative to body mass (RAM) (Table 1). Figure 4a shows representative tissue micrographs of cold- and warm-acclimated trout atria, stained with H&E, where the highly trabeculated and thin-walled chamber morphology is evident. There was no significant difference in myocyte bundle cross-sectional area with temperature acclimation (Fig. 4b); however, the EBS space between myocyte bundles was increased by 32% with cold acclimation and decreased by 29% with warm acclimation compared to controls (*P* < 0.05; Fig. 4c). This suggests that more myocyte bundles are present in the warm tissue but that their individual size does not change.

Tissue growth with warm acclimation was supported by an 8.8-fold increase in vascular endothelial growth factor (VEGF) compared to the cold (*P* < 0.05; Fig. 4d), but there was no difference in the expression of a marker of hyperplasia, proliferating cell nuclear antigen (PCNA; Fig. 4c). mRNA expression of cardiac muscle-specific growth genes did vary with thermal acclimation. Ventricular myosin heavy chain (VMHC) and small myosin light chain 2 (SMLC2) were 2.8- and 2.9-fold lower in the cold- than in the warm-acclimated group, respectively (*P* < 0.05; Fig. 4c). This would be expected to promote muscle growth during warming. However, another marker of muscle growth, muscle LIM protein (MLP) expression, was not different between cold- and warm-acclimated animals; both were depressed compared with controls (Fig. 4c).

Regulator of calcineurin (RCAN1) activates the calcineurin-NFAT signalling cascade which promotes hypertrophic growth [64, 78, 84, 31, 35]. RCAN1 mRNA expression was 2.7-fold lower in the cold- compared to the warm-acclimated atrium (*P* < 0.05; Fig. 4e). Atrial natriuretic peptide (ANP) and brain natriuretic peptide (BNP) are released by cardiomyocytes in response to stretch caused by chronic pressure or volume overload and associated with activation of the foetal gene programme [38, 74]. ANP mRNA expression following chronic cooling was 6.4-fold lower than that in warm-acclimated atria (*P* < 0.05; Fig. 4e). Temperature acclimation did not alter mRNA expression of BNP (Fig. 4e).

## Discussion

The fish heart remodels with seasonal temperature change [23, 36, 39, 40, 11, 13, 19, 43, 42, 30]. Here, we focused on the passive properties of the rainbow trout atrium across multiple levels of biological organisation following chronic cooling and warming. Based on our knowledge of fish ventricle remodelling [23, 39, 40, 13, 19, 36] and human atrial remodelling [26, 59, 10], we hypothesised that chronic cooling would increase atrial stiffness and fibrosis and upregulate growth factors associated with pathological remodelling in mammals, and that the opposite would occur following warming. We found that cold increased passive stiffness of the whole atrium and micromechanical stiffness of tissue sections. Increased stiffness was associated with an upregulation of collagen-promoting genes. Conversely, warming moderately increased whole atrial compliance, collagen-degrading genes were upregulated and gelatinase activity of collagen-degrading MMPs was increased. Thus, we can accept the first and second hypotheses that compliance and fibrosis increased in the cold and decrease in warm in trout atria. The effect of thermal acclimation on atrial muscle growth was more ambiguous. Cold acclimation increased EBS and reduced mRNA expression of some but not all, muscle-specific and hypertrophic growth markers. When changes did occur with chronic cooling, the opposite responses occurred following chronic warming. Aspects of this remodelling phenotype reflect mammalian pathological atrial remodelling, which is often associated with chronic dilation and increased myocardial stiffness [79, 60]. Therefore, the trout heart may be an interesting vertebrate model for investigating the reversibility of chronic dilation and stiffening of the atrium.

### Thermal remodelling of atrial compliance

Although ex vivo atrial pressure-volume filling curves have previously been generated for fish [18, 51], this is the first study where they have been used to probe atrial remodelling. Temperature acclimation significantly altered atrial pressure-volume relationships. Cold-acclimated chambers were stiffer and warm-acclimated chambers were more compliant than controls when all were tested at a common temperature over a range of filling volumes [12, 23, 25]. The range of volumes used here encompasses stroke volume measurements from in situ heart studies for trout, and although central venous pressure in trout can be low (< 0.1 kPa in the sinus venous of resting trout), maximal mechanical efficiency of the trout heart has been observed at a preload of ~ 0.3 kPa [23, 25]. Micromechanical stiffness of the atrium also increased with cold; however, no difference was found between the mean *E*_r_ between control and warm-acclimated groups. The increased micromechanical stiffness suggests that cold temperature leads to a remodelling of tissue ultrastructure and/or matrix organisation, which is exerting a functional effect on the myocardium. The distribution of *E*_r_ accumulative frequency curves suggests that mechanical remodelling following thermal acclimation is due to homogenous structural and/or compositional remodelling of the whole atrial tissue, rather than isolated or specific regions of the tissue. This suggests that cooling does not result in excess stiff material but rather a lower proportion of compliant material. At lower *E*_r_’s, changes in intrinsic myocyte stiffness could also contribute to the micromechanical changes observed here [61].

### Thermal remodelling of atrial extracellular matrix

In mammals, pathology-driven remodelling of the atria is often associated with atrial fibrosis [79]. In fish, the collagen surrounding the trabeculae of the fish atrium is thought to support the architecture of this thin-walled chamber [28]. We did not detect significant fibrosis in the fish atria following chronic cooling despite changes in overall chamber stiffness. Our histological analysis may not have enough resolution to resolve endomysium collagen (i.e. surrounding and interconnecting individual myocytes and capillaries) and thus may be limited to perimysium collagen (i.e. surrounding and interconnecting myocyte bundles). We did observe a trend (*P* = 0.063) for increased collagen deposition with cold acclimation which is supported by the significant changes in the fish-specific collagen gene Col1a3. Col1a3 expression was strongly upregulated following chronic cooling and downregulated following chronic warming. Thus, increased atrial stiffness in the cold may be associated with an increase in the fish-specific collagen col1a3. There may be qualitative changes in collagen content from col1a2 and/or col1a2 to the fish-specific col1a3.

The increased gelatinase activity of MMPs following warm acclimation suggests a reduction in collagen degradation, which was supported by increased mRNA expression of MMP9. Moreover, mRNA expression of MMP2, MMP9 and MMP13 was reduced in the cold-acclimated animals compared to control. In fish, MMP13 catalyses the hydrolysis of collagen, degrading it to gelatin [27] and MMP2 and MMP9 digest the gelatin into removable waste products [45]. We found no difference in the mRNA expression of the pro-collagen regulatory enzyme TIMP2 following thermal acclimation which differs from our previous finding in the ventricle [35]. TIMPs other than TIMP2 may regulate the ECM in the fish atria. It is difficult to marry the weak fibrosis in the cold atrium assessed via histology with the clear changes in passive stiffness, gene transcripts and enzyme activity. We suggest different analysis of collagen (hydroxyproline), in situ hybridisation or investigating different signalling molecules may shed light on this. For example, we did not investigate the role of transforming growth factor beta (TGF*β*) in modulating ECM in this study but previous work has shown correlations between TGF*β*, MMP expression and col1a1 in rainbow trout [69] and zebrafish [6].

We also did not find any evidence of elastin in the rainbow trout atrium using Miller’s elastic stain. This differs from mammalian atria [75] and some previous data from the goldfish atrium where elastin fibres were visualised using orcein stain [20]. In addition, non-ECM components of the myocardium may alter atrial compliance following temperature acclimation, such as the titin [58] and the actin cytoskeleton [61], which could alter intrinsic stiffness of the cardiac myocytes.

### Thermal remodelling of the working atrial myocardium

We found no difference in relative atrial mass to body mass following thermal acclimation. Previous work in fish has focused primarily on the ventricle, and in this tissue, sex, maturation and circannual rhythms influence the degree of overall hypertrophy with females and immature phenotypes showing less hypertrophy than with males and mature phenotypes [8, 19, 39, 40]. All the fish in our study were female, and thus we would suspect a limited degree of overall hypertrophy [8, 19, 39]. However, atrial myocyte hypertrophy following chronic cooling has been observed in a mixed sex population [80]. We found an increase in EBS following cold acclimation and a decrease in EBS following warm acclimation. When interpreting changes in EBS, it is important to consider that the atrial tissue was fixed flaccid and after bisecting and thus distention was not held constant between preparations by pressure but rather by the relaxed state of the tissue, which was promoted by suspension in Ca^2+^-free saline before fixation. Changes in EBS occurred without a significant change in myocyte bundle cross-sectional area, suggesting it may be due to increased cardiac preload increasing inflation of the atrium. This result is supported by our mRNA expression data that shows muscle-specific growth factors (MLP and SMLC2) and a marker of angiogenesis (VEGF) was reduced following cold acclimation, while SMLC2 and VEGF were increased following chronic warming. These findings differ from an earlier transcriptomics study on trout atrial tissue following cold acclimation [71] where atrial myocyte size increased alongside markers of muscle growth. Reasons for the discrepancy are not known but may include differences in sex and maturational status of the trout [23, 39, 40, 13, 19, 36].

Increased VEGF expression with warm acclimation in the current study may be required to increase blood supply as water oxygen content is reduced as water temperature rises, and angiogenesis is also associated with hypertrophic growth in mammals [81]. In mammals, chronic dilation increases EBS which can be further supplemented by apoptosis, reducing myocyte number during pathology [79, 21, 84]. While we did not probe for apoptosis in this study, apoptosis has been shown in the fish heart following remodelling induced by exposure to angiotensin II [29]; thus, it may be important to investigate this in future work. Atrial dilation in mammals can lead to AF [55]; however, we did not address whether fish exhibit AF in vivo following cold acclimation.

**Fig. 5:**
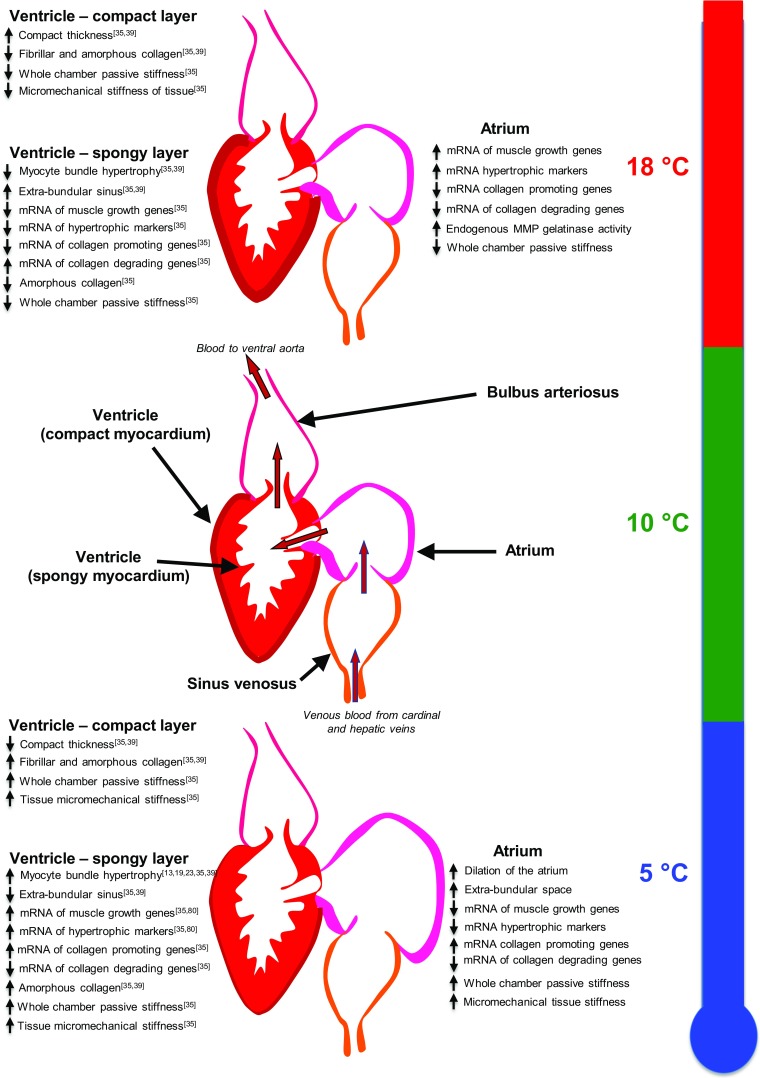
An overview of atrial and ventricular remodelling in rainbow trout exposed to chronic cold (5 °C) and chronic warm (18 °C) temperature. Superscript numbers refer to the reference list for the ventricular data. Atrial data summary is from the current study

Upregulation of ANP mRNA expression in the warm-acclimated atrium suggests an increase in stretch or myocyte hypertrophy which may appear inconsistent with our suggestion of atrial dilation following cold-induced volume overload [63]. However, the in vivo effect of ANP on the trout cardiovascular system is vasodilatory, reducing venous return blood to the heart by increasing venous compliance, which in turn decreases cardiac output and arterial pressure [56]. Due to the long acclimation period in this study, it is possible that atrial dilation with cold acclimation reduces the need for the vasodilatory properties of ANP. Mitogen-activated protein kinases (MAPKs) and the calcineurin-NFAT pathway are central to pathological hypertrophic growth in mammals [83, 4]. In some mammalian pathologies of the atria, hypertrophic signalling cascades are activated to promote the increased protein synthesis required for hypertrophic muscle growth [48, 68, 76]. Ventricular expression of RCAN1 can increase calcineurin-NFAT signalling and enhance hypertrophic growth of myocytes in mammals and fish [49, 31, 35] (see Fig. 5); however, it has also been shown to have cardio-protective, anti-hypertrophic effects [64]. RCAN1 gene expression in the atrium during cardiac remodelling is relatively unexplored. The reasons for high levels of RCAN1 mRNA with warm acclimation, in this study, are unclear. It may be due to other stresses on the atrium [65] or due to the high ANP mRNA levels [77].

### Differences between ventricular and atrial remodelling in fish

The remodelling response of the fish atrium shares some but not all characteristics with ventricular remodelling (Fig. 5). The rainbow trout ventricle is composed of two layers: an outer compact layer that is vascularized, and an inner spongy layer whose metabolic demands are met by venous blood traversing the heart. The increase in EBS with cold acclimation we report here for trout atria is the opposite response to that seen in the trout ventricle [39, 35] (Fig. 5). Ventricular myocyte cross-sectional area increases and EBS decreases in the rainbow trout with cold acclimation [39, 35], suggesting a spongy layer-driven ventricular hypertrophy [13, 23] (Fig. 5). The differential response of the atrium and ventricle highlights the different stresses imposed by increased cardiac preload on the two chambers and the response required to maintain cardiac function. The ventricle shows a significant increase in collagen deposition in the compact layer, but similar to the atrium, we did not find this difference in the spongy layer of the ventricle [35] (Fig. 5). The mRNA expression data for collagen regulation agrees with that previously determined in the rainbow trout ventricle [35] (Fig. 5). Despite only small levels of collagen, both the atrium and the spongy ventricular myocardium showed increased stiffness of the whole chamber and micromechanical stiffness in tissue sections [35] (Fig. 5). This result shows chronic cold to have the same functional effect on the atrium as we have previously shown in the ventricle. We suggest the overall reason for remodelling in both cases is to increase ventricular pumping capacity, via the Frank-Starling mechanism, as well as protect cardiac myocytes from over inflation while pumping high volumes of viscous blood in the cold [23, 73, 35].

### Perspectives and significance

Chronic dilation and stiffening of the atria, with associated fibrosis, are hallmarks of pathological remodelling in mammals [55, 60]. In fish, atrial stiffening appears to occur without the significant fibrosis seen during aging or pathological remodelling in the mammalian heart. Therefore, increased stiffness with cold acclimation is likely to protect the myocardial wall from over inflation during the haemodynamic stress of viscous blood and high preload. It may also protect the fish heart from atrial fibrillation. The fish atrium is a variable volume reservoir responsible for altering stroke volume via the Frank-Starling mechanism. At cold temperatures, blood is viscous and stroke volume is high [23]. The atrium must resist the increased haemodynamic stress, but distend further to hold a larger volume of blood to increase ventricular filling. Interestingly, Hansen et al. [25] showed the effect of increased preload to be more metabolically costly than increased afterload in the fish heart. Due to the role of the atrium as a volume reservoir, chronic dilation may occur to store venous blood preventing ventricular preload rising above maximal efficiency [25]. Atrial filling is further increased by *vis-a-fronte* filling during ventricle contraction in some fish, where a pressure void in the pericardium distends the atrium [14]. The results of this study suggest that there may be an opposite cardiac remodelling responses in chronically warmed fish compared with chronically cooled fish. However, to directly test whether the cardiac remodelling response is ‘reversible’, cardiac function would have to be assessed non-invasively in the same animals following acclimation to both temperatures (i.e. cooling then warming and/or warming then cooling).

The collagen I gene that appeared most responsive to thermal remodelling in the current study is fish-specific (Col1a3). Collagen chains containing this domain have been shown to have greater susceptibility to heat denaturation and degradation by MMP13 than collagen chains without it [66], which may explain its malleability with chronic temperature change. If this collagen domain is driving changes in chamber compliance, it may explain why a typically pathological response in mammals occurs transiently in fish.

## Electronic supplementary material

ESM 1 (DOCX 99 kb)

ESM 2 (DOCX 42 kb)

## Abbreviations

AF: Atrial fibrillation
AFM: Atomic force microscopy
ANP: Atrial natriuretic peptide
BNP: Brain natriuretic peptide
Col1a1: Collagen I alpha 1
Col1a2: Collagen I alpha 2
Col1a3: Collagen I alpha 3
DAPI: 4′, 6′-Diamidino-2-phenylindole
EBS: Extra-bundular sinus
ECM: Extracellular matrix
GLM: General linear model
H&E: Haematoxylin and eosin
MLP: Muscle LIM protein
MMP2: Matrix metalloproteinase 2
MMP9: Matrix metalloproteinase 9
MMP13: Matrix metalloproteinase 13
NFAT: Nuclear factor of activating T
PNCA: Proliferating cell nuclear antigen
RCAN1: Regulator of calcineurin 1
RT-qPCR: Real-time quantitative PCR
SMLC2: Small myosin light chain 2
TIMP2: Tissue inhibitor of metalloproteinase 2
VEGF: Vascular endothelial growth factor
VMHC: Ventricular myosin heavy chain
