# Supplementary material for: Macro- and micromechanical remodelling in the fish atrium is associated with regulation of collagen 1 alpha 3 chain expression
Source: Pflugers Arch. 2018 Mar 28;470(8):1205–19. doi: 10.1007/s00424-018-2140-1 (PMC6060776; doi:10.1007/s00424-018-2140-1)

**Supplementary Table 1.** The specific marker genes and the primers used for quantitative real-time PCR.


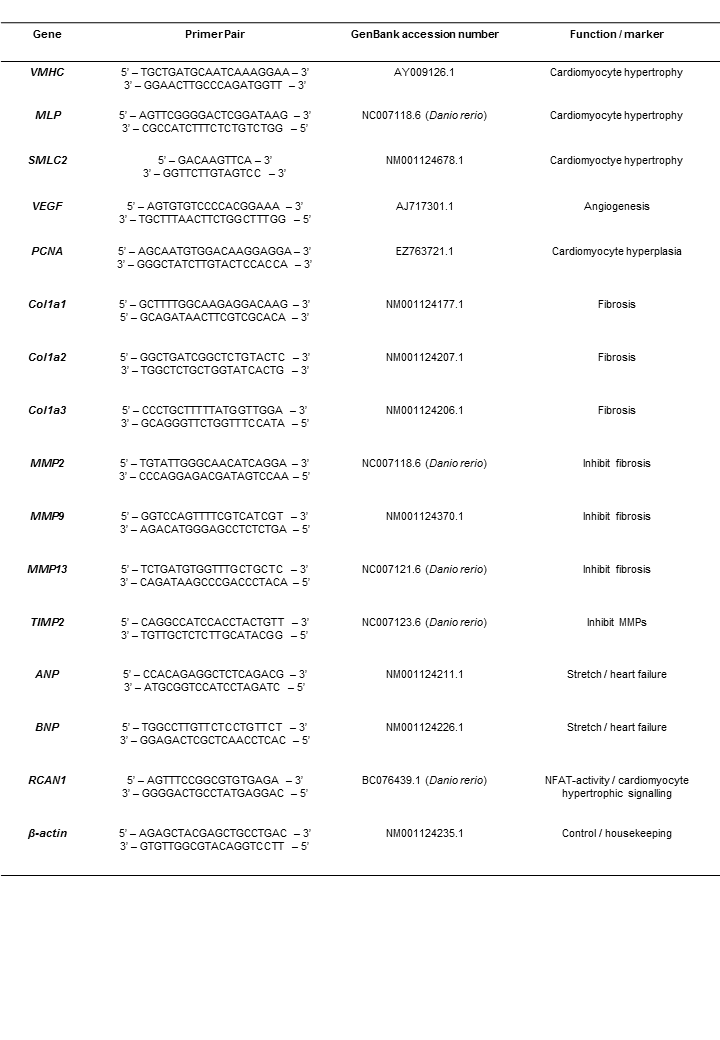

Supplement: Supplementary file 1 — (DOCX 99 kb) [file 424_2018_2140_MOESM1_ESM.docx]
