# Supplementary material for: Macro- and micromechanical remodelling in the fish atrium is associated with regulation of collagen 1 alpha 3 chain expression
Source: Pflugers Arch. 2018 Mar 28;470(8):1205–19. doi: 10.1007/s00424-018-2140-1 (PMC6060776; doi:10.1007/s00424-018-2140-1)
Supplement: Supplementary file 2 — (DOCX 42 kb) [file 424_2018_2140_MOESM2_ESM.docx]

**Supplementary Table 2.** The statistical output table from the general linear model used to assess whole chamber compliance in the atrium.


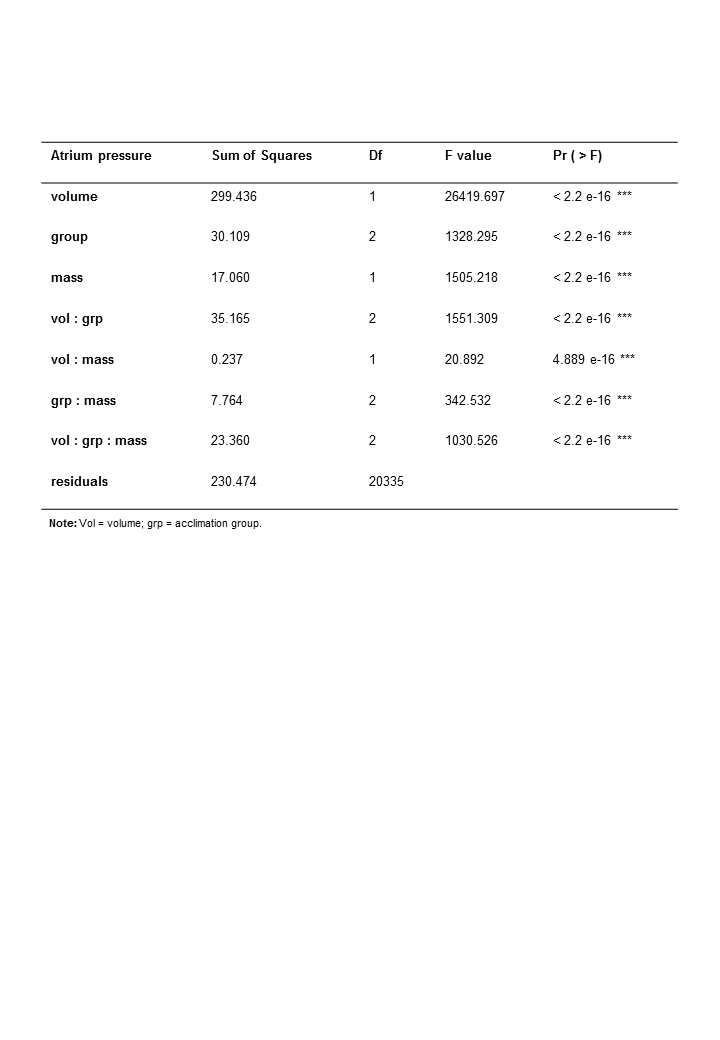


The GLM (general linear model) model used to assess statistical differences between pressure-volume curves of ex vivo hearts following thermal acclimation. The model incorporates all of the raw data across each volume and takes into account the mass and heart mass of each individual fish as a co-variate, and uses them for standardization compared to the variables of interest (here pressure and volume). This table support the statistical differences shown in Figure 1. However, for each of viewing Figure 1 only shows the pressure and volume changes, without the co-variates. Where there are 2 or more variables in the Table, it indicates a significant interaction (i.e. the effect of each interacting variable on the dependent variable depends on the value of the other interacting variable). Interactions are difficult to interpret so we have not discussed them in the test, but add to the complexity of the model so this is why they are included in the table.
